# Traditional Herbal Remedies for Burn Wound Healing in Canon of Avicenna

**Published:** 2013-11-01

**Authors:** Jale Aliasl, Fariba Khoshzaban

**Affiliations:** 1 Traditional Medicine Clinical Trial Research Center, Shahed University, Tehran, IR Iran

**Keywords:** Burns, Wound Healing, Herbal Medicine

## Abstract

Burns are a worldwide problem. The incidence of severe burns has been higher than the combined incidence of tuberculosis and HIV infections. Throughout history there have been many different treatments prescribed for burns. The Canon is the masterpiece of Avicenna’s medical books. The Canon includes a description of 785 simple drugs. Avicenna believed in burn treatment, which follows two goals. The first goal is prevention of blistering and the second goal is treatment of the burn wound after it has created blisters, cold drugs are suitable for the first goal and dry drugs with moderate in cold and hot qualities are better for second goal, this study reviewed remedies for burn wounds in Canon.

## 1. Book Information

Avicenna, The Canon of Medicine. New York: AMS Press; 1973. ISBN: 0-404-11231-5. Number of Pages: 641.

## 2. Introduction

Since man first learned to make fire, burns and scalds have been one of the most common of his injuries (1). Despite of progress in medical science, the worldwide incidence of burn injuries remains high (2). The incidence of severe burns was nearly 11 million people and was ranked fourth in all injuries, higher than the combined incidence of tuberculosis and HIV infection in 2004 (3). Throughout history there have been many different treatments prescribed for burns, although they often do not have sufficient evidence to support their use (4). This paper reviews herbs used as burn wound remedies in the canon. Avicenna's Canon of Medicine is one of the important medical classics in the world’s medical history. It was regarded as the medical guide in Europe, Arab countries and Northern African countries for a long time (5). Ibn Sina (980-1037 AD) is the great Persian physician best known in the West as Avicenna (6). The Canon of Medicine (al-Qanun-ﬁ-al-tibb) is the masterpiece of Avicenna’s medical books. Indeed, Ibn Sina’s Canon remained the most popular medical textbook in the world over the subsequent six centuries.

### 2.1. Canon

Ibn Sina divided his Canon of Medicine into five books. The first book concerns basic medical and physiological principles as well as anatomy, regimen and general therapeutic procedures. The second book is on medical substances, arranged alphabetically, following an essay on their general properties. The third book concerns the diagnosis and treatment of diseases specific to one part of the body, while the fourth covers conditions not specific to one body part, such as poisonous bites and obesity. The final, fifth, book is a formulary of compound remedies (7, 8). The Canon was used as a reference, a teaching guide and a medical textbook until well into the 19^th^ century, longer than any other medical work (9) in the schools of Europe (10). Ibn Sina began the second book on simple drugs, or material medics with a discussion on the nature and quality of drugs or temperament (they were each assigned a pair of qualities, cold or warm, dry or moist), and the way mixing them influences their effectiveness. It included a description of 785 simple drugs (6). Drugs have mineral, herbal or animal origin (11). The vegetable or herbal drugs include leaves, roots, seeds, branches, flowers, fruits and gums. The drugs have specific temperament or Mizaj.

Mizaj is an Arabic word. In the English language, the word temperament is used to describe Mizaj, which is derived from the Latin word ‘Tempero’ meaning to mix together. Mizaj plays an important role in the maintenance of health and prevention of disease. Temperament (Mizaj) has an important place, and forms the basis of pathology, diagnosis and treatment (12). The body and everything else is made up of four basic elements i.e. earth, air, water and fire which have different temperaments i.e. cold, hot, wet, and dry. After mixing and interaction of the four elements a new compound having new temperament comes into existence i.e. hot wet, hot dry, cold wet and cold dry. The body has simple and compound organs, which gain their nourishment through four humors i.e. blood, phlegm, yellow bile, black bile. The humor also assigned temperament as blood is hot and wet, Phlegm is cold and wet, yellow bile is hot and dry and black bile is cold and dry. In the treatment of disease it is of prime importance that one should be aware of the mizaj of organs in health and disease. Mizaj of a person is greatly influenced by, occupation and environment etc. The imbalance in body temperament and humors leads to the onset of disease. Therefore, treatment is based on the correction of temperament and humors to achieve a balanced state. So, the drug used for the treatment should possess, an opposite temperament from that of the diseased. A disease, which is hot in nature, can be cured by a drug, which has cold temperament and so on (13). The potency of the drug should be equal to the strength of the disease. If some of the drugs are inadequate with regard to heat when compared to the coldness of an illness, they will not be able effect a cure (7).

## 3. Objectives

This project evaluated the influence of medicinal plants on burn, and this article covers a section of the finding. To our knowledge, it is the first article in our country, which concerns this subject.

## 4. Section 1

In this study the Arabic Canon was searched for the Arabic term of “Hargh” meaning burn, for herbal drugs which are effective on burn wound healing. Next, the term was rechecked in the English translated Canon [published in the Jamia Hamdard University in New Delhi, 1998] (11) and their scientific names was adjusted using books of Iranian popular medicinal plants (by Gholamreza Amin) (14).

## 5. Section 2

Ibn Sina began the second book (on simple drugs, or material medics) with a discussion on the nature and quality of drugs or temperament (they were each assigned a pair of qualities, cold or warm, dry or moist). In the forth book, he explained kinds of injuries and wounds and their treatments. He also explained fire burns (Hargh-al nar) and scalds (Hargh- al maol har) and their remedies. He believed that burn treatment show follow two goals. The first goal is prevention of blistering and the second goal is treatment of burn wounds after blisters have developed (15). He believed that cold drugs are suitable for the first goal and for the second goal detergent and dry drugs with moderate cold and hot qualities are better. After searching between 785 simple drugs in the second book of Canon, 28 herbal drugs which are effective on burn wound healing were obtained. Their name in Canon, common name and scientific name and their temperament and their use for burn is classified in two tables. Some of them have cold temperament and they are useful for the first goal in burn remedy, prevention of blistering, and also reducing pain (Table 1), and some other herbal drugs are effective after blistering, useful for burn wound healing (Table 2).

**Table 1.** Herbal Drugs for Prevention of Blistering

Name in Canon	Common Name	Scientific Name	Temperament	Effect on Burn
**AS**	Myrtle	*Myrtus communis *L.	Its coldness is in the first degree and dryness in the second degree.	When used with olive oil, it gives relief to hot inflammations, wounds of the palms and burns. If its fruits is cooked with wine and turned into a plaster, cure burns and prevents blistering. Qayruti (a form of ointment) that is made from its ash acts similarly.
**Aqaqia**	Wild gum arabic tree	*Acacia Arabica *	If Aqaqia is obtained by the processes of washing, it is cold and dry in the second degree and if not washed, it is cold in the first and dry approximating to the third d*e*gree.	It is useful for fire burns and hot swellings when mixed with egg white.
**Dulb**	Oriental plane tree	*Platanus orientalis*	The bark and the nut of oriental plane tree is highly desiccant and is cold in the first degree.	Decoction of the bark with vinegar is useful in burns.
**Faufal**	Betel nut	*Areca catechu *L.	It is cold and dry in the third degree.	It is good for hot and hard swellings.
**Hashishah al-zujaj**	Common Pellitory	*Parietaria off*	It is cold and dry in the second degree	It dissolves swellings and its leaves cure erysipelas, fire burns and phlegmatic swellings.
**Hinna**	Henna	*Lawsonia inermis *L.	Henna is cold in the first degree and dry in the second degree	The decoction of Henna is used as a douche in burns.
**Inab al-thalab**	Garden night shade	*Solanum nigrum *L.	It is cold in the first and dry in the second degree. A variety; possessing local sedative property is cold and dry in the second degree.	It serves as a good plaster for use in hot internal or external swellings.
**Khubbazi**	Jews mallow	*Malva sylvestris *L.*, Malva neglecta *Wallr*.*	It is cold and moist in the first degree	The leaves of wild Jews mallow with olive oil are useful in burns. Similar action is attributed to its douche
**Khal**	Vinegar	*Acetum vinegar*	Vinegar is made of hot and cold substances. Both components are tenuous but the coldness is dominant. The vinegar, which is pungent, is hot while that which is not so pungent, is moist and cold. The coldness of vinegar is reduced by boiling. Vinegar is a strong desiccant.	Local application is useful in creeping ulcers, scabies and ringworms and heals burns more quickly than any other drug.
**Kafur**	Camphor	*Cinnamomum comphora *(L.) N. et Ebern	It is cold and dry in the third degree.	It prevents hot swellings.
**Kuzbara**	Coriander	*Coriandrum sativum *L.	It is cold from the last phase of the first degree up to the second degree. It is dry in the second degree. The excessive use of its extract may be fatal due to its cooling properties.	It is useful in hot swellings when applied with white lead and vinegar.
**lisan al-hamal**	Great plantain	*Plantago major *L.	Its root is quite dry and contains less moisture. Cold effects are not sufficient to produce a numbing action. Its dryness is not intense as to cause irritation. It is an ideal drug for ulcers.	It is good for hot swellings, burns, herpes and urticaria
**Sandal**	Sandal wood	*santalum *sp.	It is cold from the last phase of the second degree to the third degree. It is dry in the second degree.	Sandal wood, particularly its red variety dissolves hot swellings
**Ward**	Rose	*Rosa centifolia *L.*, Rosa damascena *Mill.	It may be cold in the first degree, particularly in its dried state, to be cold in the first phase or the second degree.	Application of a plaster prepared from a decoction of powdered flowers without squeezing, dissolves hot inflammations. It is useful for treating ulcers. It promotes granulation in chronic ulcers.
**Zaitun (al-zait)**	Olive (oil)	*Olea europaea *L*.*	Oil of mature olive is moderately hot and somewhat moist and when treated with water it becomes moderate in moisture content and dryness as it also grows less hot. In short, the ripe variety of olive is hot while its oil is moderately moist. The unripe olive is cold, its bark and leaves are also cold.	Local use of Zaitun-al-ma treated with water and salt, prevents formation of blisters in burns.

**Table 2.** Herbal Drugs for Treatment of Burn Wound After Blistering

Name in Canon	Common Name	Scientific Name	Temperament	Effect on Burn
**Abu Khalsa**	Dryers bugloss	*Arnebia euchroma* (Royle) I. M. Johst	According to Galen, its temperament is hot and dry while some other physicians hold an opposite opinion.	It is applied with wax and olive oil on the wound, especially on burn wounds
**Adas**	Lentil	*Lens esculenta Moench*	It is moderate in hotness and dryness or inclines towards hotness.	Application of a plaster prepared by cooking with vinegar dissolves scrofula and hard swellings. Decoction with vinegar heals deep ulcers and reduces or stops their discharge.
**Ghaliyun**	Aghailion	*Galium verum*	It is hot in the first and dry in the second degree	The flowers and leaves of this plant are useful for burns.
**Hiofariqun**	Hypericon	*Hypericum perforatum *L.	It is hot in the second degree and dry in the last phase of the second degree.	A plaster of its leaves is useful for healing burns, large wounds and malignant ulcers.
**Hulbah**	Fenugreek	*Trigonella foenum–graecum *L.	It is hot in the last phase of the first degree and dry in the first degree	Administration with rose oil is useful for burns.
**Kundur**	Frankincense	*Boswellia carterii *Bird	It is hot in the second and desiccant in the first degree. Its peel is desiccant approximately in the third degree.	It is applied with swine fat on burn ulcers and cold- fissures. It heals the ulcers caused by burns.
**Lablab**	Lablab	*Dolicus lablab*	It is moderate but somewhat hot, dry and a softening drug	Healing is achieved when its fresh leaves are decocted and given with wine. It is applied as a plaster, particularly in the form of a Qairuti in cases of burns.
**Narjis**	Narcissus	*Narcissus tazzeta*	It is hot and desiccant in the third degree	It is applied in powdered form with honey to burns.
**Sanobar**	Pine	*Pinus *sp.	It is hot and dry in the third degree	The barks of Sanobar are useful in for burning ulcers.
**Sarish**	Asphodel	*Eremurus persicus *(Jaub.& Soach) Boiss.	It is hot in the first degree.	Its plaster is useful for burns.
**Sausan**	Lily	*Iris *spp	The white Lily known as Sausan Azad is hot and dry in the second degree. Wild iris root is highly warming and desiccant. The root of the plant is useful for hot- water burns owing to its desiccant and moderately detergent nature.	The cultivated variety is the most useful drug for treating burns and scalds.
**Silq**	Beet	*Beta vulgaris*	It is hot and dry in the first degree, but in fact, it possesses composite properties. Some other physicians consider it to be cold but there is no doubt about its moist nature.	Its boiled leaves are good burns.
**Simsim**	Sesame	*Sesamum indicum *L.	It is hot in the middle and moist in the last phase of the first degree.	Sesame is applied on burns.
**Zaitun (al-zait)**	Olive (oil)	*Olea europaea* L.	Oil of mature olive is moderately hot and somewhat moist and when treated with water it becomes moderate in moisture content and dryness as it also becomes less hot. In short, the ripe variety of olive is hot while its oil is moderately moist. The unripe olive is cold, its bark and leaves are also cold.	Local use of Zaitun-al-ma treated with water and salt, prevents formation of blisters in burns.

## 6. Section 3

Ibn Sina, is one of the most famous Persian physicians and Scientist in Iran, and in the world and Canon is his medicinal book. Although there are many studies on Avicenna and his Canon, this is the first study focusing simple herbal drugs in Canon for burn wound healing. In this study second book of Canon was searched for burn remedies, and 28 simple herbal drugs take. Avicenna described burn treatment following two goals. The first goal is prevention of blistering, and the second goal is treatment of burn wound after blister formation (15). He believed; cold drugs are suitable for the first goal (Table 1) and for the second goal detergent and dry drugs with moderate in cold and hot qualities are better (Table 2). Therefore, immediately after a burn wound we must use a herbal remedy by cold temperament like “As” *Myrtus communis* L. or “Khubbazi” *Malva sylvestris* L, and when the wound has more discharge it is better to use herbal drugs by dryness temperament and so on, therefore, treatment should be selected based upon the type of wound. There are many effective remedies in Iranian Traditional Medicine for wound healing. We hope soon these herbs are clinically tested and widely available to people for burn wound treatment.
